# Pathogenesis of an experimental mycobacteriosis in an apple snail

**DOI:** 10.3389/fimmu.2023.1253099

**Published:** 2023-10-09

**Authors:** Cesar Cruz-Flores, Cristian Rodriguez, Constanza Giai, Israel A. Vega, Alfredo Castro-Vazquez

**Affiliations:** ^1^IHEM, CONICET, Universidad Nacional de Cuyo, Mendoza, Argentina; ^2^Facultad de Ciencias Exactas y Naturales, Universidad Nacional de Cuyo, Mendoza, Argentina; ^3^Instituto de Fisiología, Facultad de Ciencias Médicas, Universidad Nacional de Cuyo, Mendoza, Argentina

**Keywords:** hemocyte aggregations, hemocyte nodules, kidney, lung, gill, urate storage tissue, histopathological lesions

## Abstract

In this work, we aimed at investigating cell and tissue responses of the apple snail *Pomacea canaliculata*, following the inoculation of the zoonotic pathogen *Mycobacterium marinum*. Different doses were tested (10, 20, 65, and 100 M CFU) and the mortality rate was negligible. The histopathogenesis was followed at 4, 9, and 28 days after inoculation. Overt histopathological lesions were consistently observed after the two largest doses only. In the lung, marked hemocyte aggregations, including intravascular nodule formation, were observed within the large blood veins that run along the floor and roof of this organ. Hemocyte aggregations were found occluding many of the radial sinuses supplying the respiratory lamina. Acid-fast bacilli were contained in the different hemocyte aggregations. In addition, hemocytes were observed infiltrating the storage tissue, which makes up most of the lung wall, and the connective tissue of the mantle edge. Additionally, signs of degradation in the storage tissue were observed in the lung wall on day 28. In the kidney, nodules were formed associated with the constitutive hemocyte islets and with the subpallial hemocoelic space, in whose hemocytes the acid-fast structures were found. Electron microscopy analysis revealed the presence of bacteria-containing phagosomes within hemocytes located in the surface zone of the islets. Additionally, electron-dense spheroidal structures, which are likely remnants of digested mycobacteria, were observed in close proximity to the hemocytes’ nuclei. The size attained by the hemocyte nodules varied during the observation period, but there was no clear dependence on dose or time after inoculation. Nodules were also formed subpallially. Some of these nodules showed 2–3 layers with different cellular composition, suggesting they may also form through successive waves of circulating cells reaching them. Nodular cores, including those formed intravascularly in the lung, would exhibit signs of hemocyte dedifferentiation, possibly proliferation, and death. Hemocyte congestion was observed in the hemocoelic spaces surrounding the pallial ends of the renal crypts, and the renal crypts themselves showed de-epithelization, particularly on day 28. The diverse cellular responses of *P. canaliculata* to *M. marinum* inoculation and the high resilience of this snail to the pathogen make it a suitable species for studying mycobacterial infections and their effects on cellular and physiological processes.

## 1 Introduction

The first molluscs to be studied in relation to mycobacterial infections were gastropods. Pan (1) first reported an association of pathogenic acid-fast bacilli with a mollusc while studying the planorbid snail *Biomphalaria glabrata* (as *Australorbis glabratus*). Following these findings, Michelson’s (2) study on the planorbid *Helisoma anceps* included the first isolation of a *Mycobacterium* from an invertebrate and the first experimental mycobacterial infection in a mollusc.

More recently, Marsollier et al. (3) reported that “viable” bacilli appeared in feces after feeding an apple-snail (*Pomacea canaliculata*, Ampullariidae) with macrophytes covered with biofilms of *Mycobacterium ulcerans*, the causative agent of Buruli ulcer, the third most frequent mycobacteriosis in humans (4). However, since no growth of the mycobacteria was detected in tissues and they had disappeared 50 days after exposure, the authors concluded that *P. canaliculata* may only behave as a “passive host” for *M. ulcerans*.

In preceding studies, we reported the response of *Pomacea canaliculata* to a non-pathogenic immune challenge, the intra-hemocoelic injection of *Saccharomyces cerevisiae*, and proposed that the kidney and lung would function as immune barriers against the spread of circulating microbes, because of their position in the systemic circulation and the intricacy of their microcirculation (5). The current study was aimed to test this hypothesis of the organ barriers, but by studying the immune cell reactions to a pathogen. We chose *Mycobacterium marinum*, another water-borne mycobacterium, whose optimal growth temperature overlaps with the optimal temperature for reproduction in *P. canaliculata* (6). *M. marinum* is a phylogenetic relative of *Mycobacterium tuberculosis* and is the causative agent of a tuberculosis-like disease in fish and of skin ulcers in humans (7) and together with *M. ulcerans* and *Mycobacterium bovis* are the agents of the most prevalent mycobacterial diseases in humans.

*Pomacea canaliculata*, the experimental host in this study, is an ampullariid snail native to the Plata basin, where it inhabits a variety of freshwater habitats. It is a relatively large snail with a lifespan of 1-2 years in its natural range and can be easily bred in captivity. It has gained international notoriety due to its introduction to Southeast Asia, where it has become a threat to rice crops and an alternative host for *Angiostrongylus cantonensis*, the causative agent of eosinophilic meningitis, a zoonosis that is rarely lethal but often leads to disability. *Angiostrongylus cantonensis* has more recently spread to South America, overlapping with the native range of *P. canaliculata*.

## 2 Materials and methods

### 2.1 Apple snail species identification and culture

We used adult males and females of the Rosedal strain of *P. canaliculata*, whose origin and culture conditions we have reported (e.g., 5), as well as its nuclear genome (8). The Institutional Committee for the Care and Use of Laboratory Animals (School of Medicine, National University of Cuyo) approved the procedures for culture, experimentation, sacrifice and tissue sampling of the snails (Approval Protocol No 55/2015). All groups were composed of an approximately equal number of sexually mature females and males. They were at least 25 mm long and approximately 5 months old. All animals within each aquarium received the same treatment.

### 2.2 Bacterial culture and preparation of the inoculum

We used the “M” strain (ATCC BAA-535) of *Mycobacterium marinum*. The bacteria were grown at 30°C with slow agitation in Middlebrook 7H9 broth medium (Becton Dickinson), enriched with 0.5% albumin (SIGMA), 0.5% glycerol (SIGMA), 0.4% glucose (Biopack), and 0.25% Tween 80 (SIGMA) and was used at the mid‐ to late‐log phase (OD = 1.0). The bacteria were kindly provided to us by Professor Maria I. Colombo (IHEM-CONICET, Mendoza, Argentina) (9). Cultured bacteria were washed twice with phosphate‐buffered saline (PBS) before use. For disrupting bacterial clumps, suspensions were passed 5–10 times through a 200 μL-micropipette tip and centrifuged at 1500 rpm for 1 min. To verify the bacterial dose, *M. marinum* suspension was diluted in serial dilutions and plated on 7H10 agar (Becton Dickinson) enriched with 0.5% albumin (Sigma), 0.5% glycerol (Sigma) and 0.4% glucose (Biopack) for counting bacterial colonies.

### 2.3 Snail mortality rate after inoculation

The snails in the study were injected in the visceral hemocoel, as previously described (5), with either a buffer suitable for injection in *P. canaliculata* (PcBS, 10) or 65 or 100 million colony-forming units (M CFU) of *M. marinum*. The survival of the snails was monitored once daily for a period of 28 days following inoculation, and the experiment was replicated once. Since no apparent differences were observed, both replicates were pooled for presentation. Finally, each group consisted of 66–70 snails. To determine if an animal had died, we performed a body withdrawal test by gently pulling the operculum with forceps. If there was no response or movement, the animal was considered dead, and the soft tissues were promptly fixed in a 4% formaldehyde solution for subsequent histological examination.

### 2.4 Experimental infection with *M. marinum*

A total of 45 animals were allocated for this experiment. Inoculation was made in the visceral hemocoel as reported elsewhere (5). The inoculum was prepared by resuspending the mycobacteria in PcBS and the suspensions were adjusted to administer nominal doses of either 10, 20, 65 or 100 M CFU per snail, in an 80 μL-injection volume, which was also used for buffer-injected controls. All groups were composed by an approximately equal number of males and females and were injected either with vehicle or a single dose of the inoculum. Each aquarium (48 cm × 32 cm × 16 cm, water level ~10 cm) contained 9 animals that had received the same treatment. The animals were fed with lettuce *ad libitum* until sacrifice, at either 4, 9, or 28 days after inoculation or vehicle injection. The few animals that died were not replaced.

### 2.5 Sacrifice and histological and histochemical examination

Before sacrifice, the animals were placed in a water/ice bath (4°C) for 20–30 min to induce relaxation and minimize nociception. The shell was then carefully cracked open, and the kidney, lung, and gill (i.e., the organs regarded as immune barriers; (5), as well as the approximate site of inoculation in the digestive gland hemocoel, and the mantle border were sampled. The samples were fixed in 4% formaldehyde, dehydrated in an ethanol series, and embedded either in a paraffin mixture (Histoplast^®^, Biopack, Argentina) or in resin (Historesin^®^, Leica Microsystems GmbH, Germany). Rotary microtomes Microm HM325 and Leica HistoCore Autocut were used for cutting paraffin- and resin-embedded material, respectively. Then, some sections were stained with Harris hematoxylin and eosin (Biopack, Argentina). Strongly electronegative components of the mycobacterial wall (mycolic acid and murein) were detected by the Ziehl-Neelsen’s carbolfuchsin method. Differentiation was achieved using an ethanol-hydrochloric acid mixture, with methylene blue used as a counterstain. However, Ziehl-Neelsen’s method could only be used on paraffin sections because resin sections were invariably detached from the glass slides by the ethanol-acid mixture. Carbolfuchsin is a basic stain that binds strongly to electronegative components of the mycobacterial wall (mycolic acid and murein) and the mycobacteria are stained red.

### 2.6 Transmission electron microscopy

Kidney samples of snails injected with vehicle or 100 M CFU were dissected out and fixed in Karnovsky’s fluid (4% paraformaldehyde and 0.8% glutaraldehyde in 0.1 M phosphate buffer, pH 7.4, at 4°C). After one day, the tissues were washed thrice in phosphate buffer and transferred to 1% osmium tetroxide overnight. Then, they were rinsed in distilled water and treated with an aqueous solution of 2% uranyl acetate for 40 min, gradually dehydrated in a graded ethanol series followed by acetone, and finally embedded in Spurr resin (Ted Pella, Inc.). Ultrathin sections, obtained with a Leica Ultracut microtome, were mounted on copper grids and stained with uranyl acetate and lead citrate and examined with a Zeiss EM 900 electron microscope.

## 3 Results

First, it is important to note that understanding the impacts of inoculation on *P. canaliculata* necessitates prior comprehension of certain morpho-functional characteristics in undisturbed apple snails that are not commonly known. To facilitate this understanding, relevant details pertaining to the intact snail condition will be presented before each subsection as necessary. Moreover, **Supplementary Figures S1**-**S5** have been included to visually depict these aspects.

### 3.1 Effective doses and the mortality rate after inoculation

Two experiments were conducted. The first aimed to determine effective doses for inducing histopathological lesions, and these animals were also used to morphologically describe those lesions. The second experiment focused solely on recording survival of the inoculated snails, and histological examination was performed only on necropsy material.

In the first experiment, overt histopathological lesions consistently occurred following inoculation with doses of 65 or 100 M CFU of *M. marinum*. This paper will focus on describing the effects of these doses. However, it is noteworthy that one out of six animals exhibited a response to a lower dose (20 M CFU), and that no responses were observed to vehicle injection (9 animals) or to a dose of 10 M CFU (6 animals).

In the second experiment, only nine deaths were recorded throughout the entire 28-day observation period, out of the 206 injected snails (**Table 1**). A single animal died after vehicle injection in the control group. Also, two snails (2.8%) died after inoculation with 65 M CFU, and six snails (8.5%) died after inoculation with 100 M CFU. Necropsies conducted on the deceased animals revealed a decay or complete lysis of storage cells (Section 3.2.2) in the lung (not shown in pictures). The surviving animals appeared less active but consumed the usual amount of lettuce (about 170 mg/kg of drained live mass, 10). Although copulations and oviposition episodes occurred, their frequencies were not recorded.

**Table 1:** Snails’ deaths during the 28-days period after vehicle injection or *M. marinum* inoculation.

	Vehicle	65 M CFU	100 M CFU
Total number of snails injected	66	70	70
Total number of dead snails	1	2	6
Days when snails died	12	8, 12	7, 7, 7, 8, 16, 27
Percent of dead animals	1.5	2.8	8.5

### 3.2 The lung and the gill

The lung is a flattened sac-like, heavily vascularized organ. The main lung veins exhibit thick fibromuscular walls and run along the roof and the floor of the lung sac and, in turn, give rise to smaller perpendicular sinuses, the ‘radial sinuses’ (5), which supply the ‘respiratory lamina’ (i.e., the part of the lung involved in gas exchange with air; see also **Supplementary Figures S1**, **S2**).

#### 3.2.1 Aggregations within hemocoelic sectors in the lung

**Figure 1:**
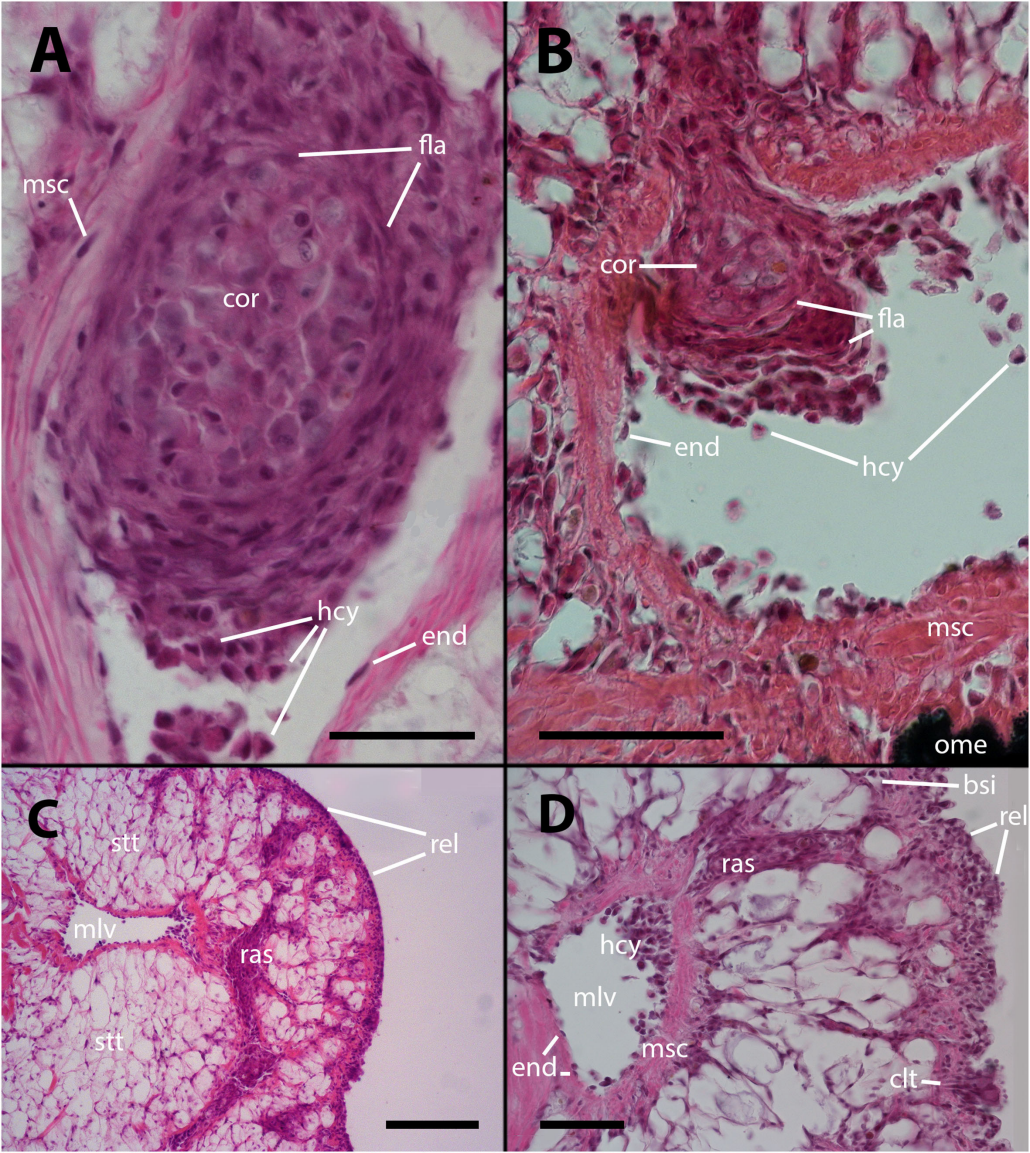
Intravascular hemocyte aggregations in the lung of *P. canaliculata* after *M. marinum inoculation*. **(A)** A large intravascular nodule surrounded by flattened cells containing mature hemocytes, dedifferentiated hemocytes, and some anucleated masses of dead cells. The main lung vessels, including the vessel wall, exhibit muscular walls and an endothelium-like lining. Sacrificed 9 days after inoculation with 100 M CFU. **(B)** A large intravascular nodule surrounded by flattened cells, and with a distinct core, being propelled into one of the radial sinuses and causing obstruction. Sacrificed 9 days after inoculation with 100 M CFU. **(C)** Hemocyte congestion obstructing the radial sinuses and respiratory lamina. The sinuses arise from the main vessels with fibromuscular walls and endothelial linings. The congestion extends beyond the eosinophilic connective layer underlying the sinuses of the respiratory lamina, which are also filled with hemocytes. Sacrificed 4 days after inoculation with 100 M CFU. **(D)** View at higher magnification showing the fibromuscular wall of one of the main vessels and its discontinuous endothelial lining, while some hemocytes are still contained within the vessel. The obstructed radial sinuses and respiratory lamina are also visible. Two details of the respiratory lamina are indicated: a single sinus that contains two floating hemocytes while all other sinuses are obliterated (right upper corner) and a ciliary tuft displaying its roots and some basophilic clumps retained by its cilia (right lower corner, see **Supplementary Figures S1**, **S2**). Sacrificed 4 days after inoculation with 100 M CFU. Abbreviations: bsi, blood sinus; clt, cellular tuft; cor, cell core; end, endothelial cell; fla, flattened cells; hcy, hemocytes; mlv, main lung vein; msc, muscle cells; ome, outer mantle epithelium; ras, radial sinus; rel, respiratory lamina; stt, storage tissue. Scale bars: **(A)** 25 µm; **(B)** 50 µm; **(C)** 100 µm; **(D)** 50 µm. Unless otherwise indicated, this and all subsequent figures were obtained from paraffin-embedded material and stained with hematoxylin-eosin.

The histological features of the lung of vehicle injected animals did not differ from previous descriptions. (5; see also **Supplementary Figures S1**–**S2**, 11). However, after inoculation with either 65 or 100 M CFU, large hemocyte aggregates form inside the main lung veins (**Figure 1A**). Frequently, these aggregates appear loose within their lumina, and some are propelled toward the origin of radial sinuses and may obstruct them (**Figure 1B**). All large aggregates remain within the lumen and create layers surrounding a core that exhibits variable composition. This core consists of mature hemocytes, including hyalinocytes and granulocytes (12), as well as hemocytes that have undergone transformation in which their small hyperchromatic nuclei have become large and clear with prominent nucleoli, suggestive of dedifferentiation. Additionally, remnants of dead cells may be present within the cellular core (**Figures 1A, B**).

Also, it is common to observe radial sinuses filled with hemocytes in inoculated animals, but these cells are mostly not derived from aggregates in the main veins. Rather, they appear to be local aggregations of free hemocytes brought there by the blood flow (**Figures 1C, D**). In fact, most hemocyte aggregates in the radial sinuses have no continuity with any large aggregate in the main veins. Although lung lesions following inoculation are mainly intravascular, some large hemocyte aggregates may also expand into tissue, usually including portions of the respiratory lamina (**Figures 1C, D**).

**Figure 2:**
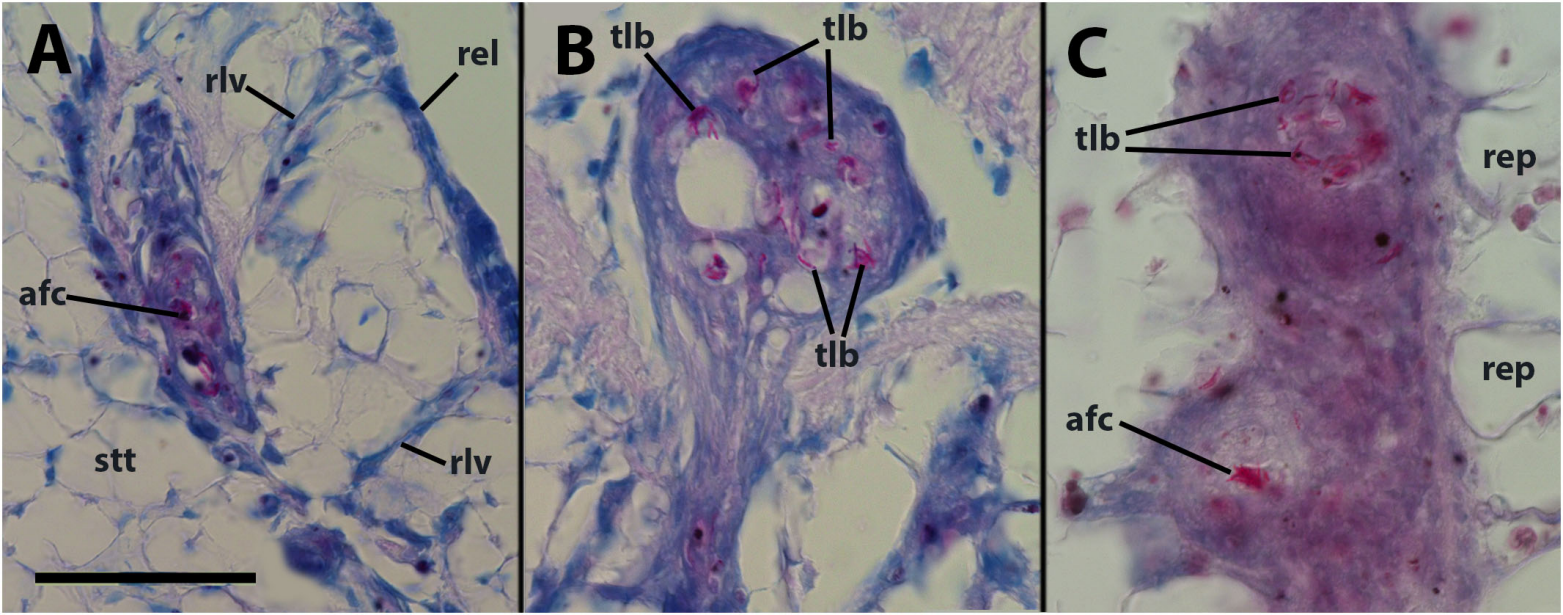
Thread-like structures and clumps of an acid-fast material within hemocyte aggregations in the lung and kidney. **(A)** Decaying cells and acid-fast clumps occluding a small lung vessel. Sacrificed 4 days after inoculation with 65 M CFU. **(B)** Numerous acid-fast bacilli in an hemocyte plug obstructing a lung vessel (similar to that in **Figure 1B**). **(C)** Two hemocyte nodules within a renal islet. Acid-fast thread-like bacilli predominate in the upper nodule, while a bulky acid-fast clump is found in the core of the lower one. afc, acid-fast clump; rel, respiratory lamina; rep, renal epithelium; rlv, radial lung vessel; stt, storage tissue. Scale bar represents 50 µm for all panels.

Within the hemocyte aggregations in lung vessels, Ziehl-Neelsen’s preparations showed the occurrence of thread-like, acid-fast bacilli as well as more bulky acid-fast clumps that are likely mycobacterial remnants (**Figures 2A, B**).

#### 3.2.2 Decay of the storage tissue of the lung wall and hemocyte infiltration

The storage tissue that makes up most of the lung walls is composed of large, polygonal cells with small and clear nuclei with no apparent nucleoli, and it accumulates uric acid, an important metabolite in maintaining oxidative balance in different physiological states (e.g., 13–15).

**Figure 3:**
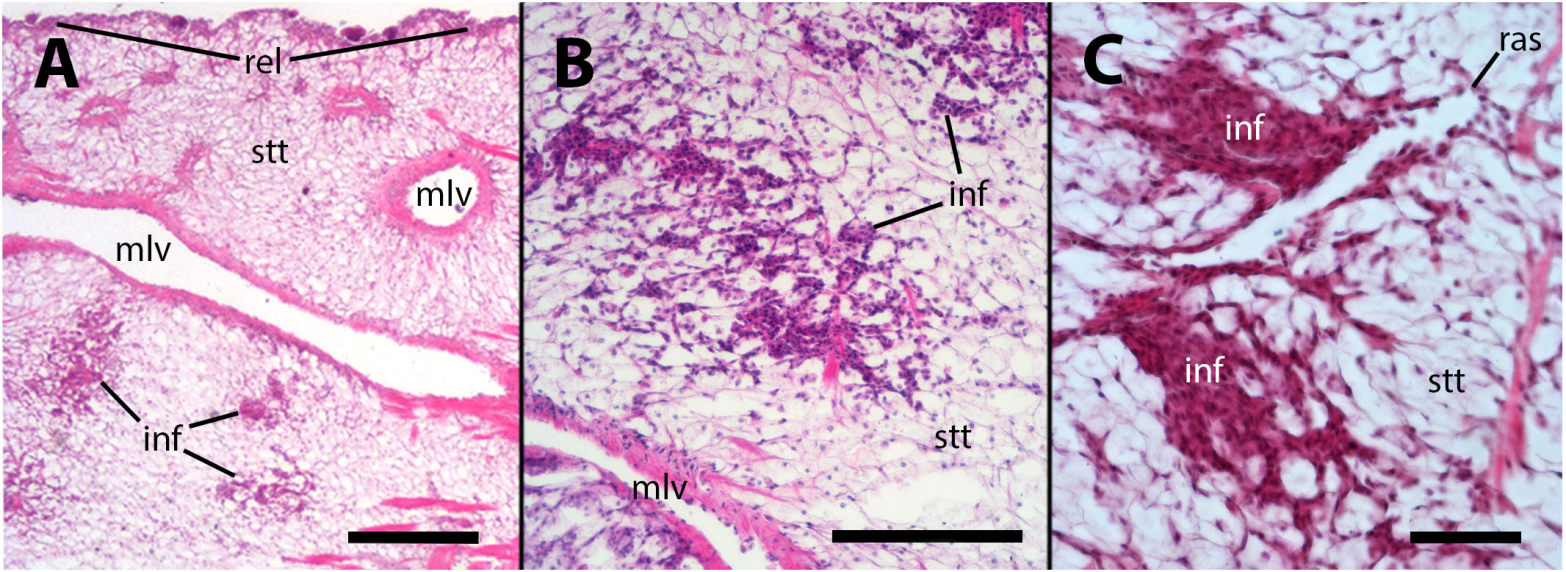
Hemocyte infiltration of the lung floor. **(A)** Field view of a tangential section showing some of the main vessels and a thinning of the storage tissue, together with some areas of hemocyte infiltration; **(B)** An area of decaying storage cells with hemocyte infiltration; **(C)** Another area of decaying storage cells at higher magnification, with more densely packed hemocyte infiltration. inf, hemocyte infiltration; mlv, main lung vein; ras, radial sinus; rel, respiratory lamina; stt, storage tissue. Treatments: **(A, B)** sacrificed 28 days after 65 M CFU inoculation; **(C)** sacrificed 9 days after 100 M CFU inoculation. Scale bars: **(A)** 500 µm; **(B)** 150 µm; **(C)** 50 µm.

No apparent changes followed vehicle injection. But, in animals inoculated with either 65 or 100 M CFU, there was some evidence of decay of this storage tissue, together with hemocyte infiltration, 28 days after inoculation or even earlier (**Figure 3**). It is worth noting that in necropsies of animals that died prior to the scheduled sacrifice, there was evidence of decay or complete lysis of urate cells in the lung wall. However, since the snails were revised only once daily, it was not possible to discern whether this was the cause or the effect of death.

#### 3.2.3 Changes in the gill epithelium

The gill has been proposed as a potential third organ barrier, along with the lung and kidney islets, due to its position in the circulation and to the presence of a semicontinuous row of intraepithelial granulocytes in the gill leaflets, near the basal membrane (16; see also **Supplementary Figures S3**, **S4**). Although the hemocoelic sinuses of the gill leaflets create regions of slow blood flow, which facilitates gas exchange, it also increases the likelihood of contact between hemocytes and the inoculated mycobacteria. However, only small nodules were occasionally formed in the leaflet sinuses after inoculation.

**Figure 4:**
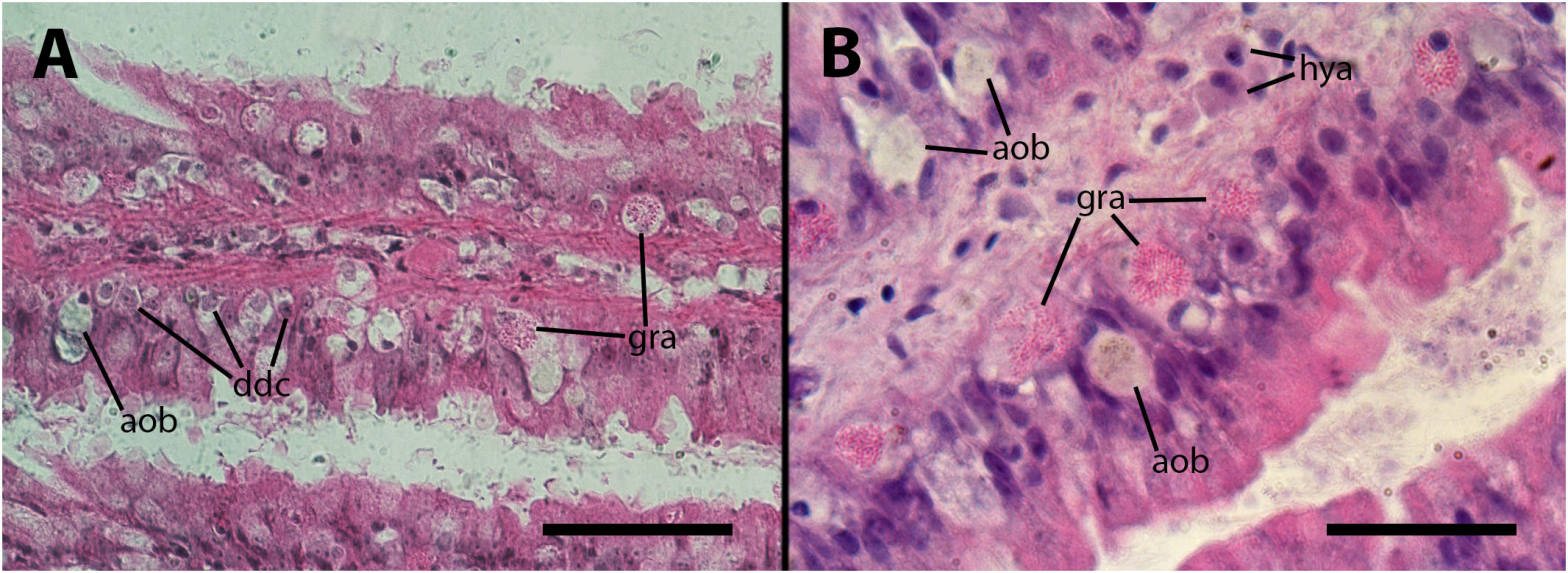
Epithelium of the gill lamellae after inoculation. In both the **(A, B)** panels, it can be observed that some granulocytes have undergone dedifferentiation or have been degraded and died, leaving behind brownish/grayish bodies. Also, morphological variation of granulocytes’ granules, including the normal rod-like granules [R-granules (12)], can be observed in both panels. Notice the large cytoplasm of some hyalinocytes in the leaflet hemocoel. aob, anucleated ovoid bodies; ddc, dedifferentiated cells; gra, granulocytes; hya, hyalinocytes. Treatments: **(A, B)** 9 days after inoculation with doses of 65 or 100 M CFU, respectively. The micrograph on **(B)** only was taken from resin embedded material. Scale bars: **(A)** 50µm; **(B)** 25 µm.

The inoculation had a disruptive effect on the barrier created by the granulocytes. This disruption was characterized by the death of numerous granulocytes and their subsequent transformation into anucleated ovoid bodies. Additionally, many of the remaining granulocytes exhibited varying degrees of distortion in their cytoplasmic granules. As it occurs in the lung vasculature, a considerable proportion of granulocytes underwent a likely process of dedifferentiation, indicated by the transformation of their small hyperchromatic nuclei into large, clear nuclei with prominent nucleoli. All these changes are illustrated in **Figures 4A, B**. We could not establish any relation with the dose employed or the time after inoculation, in either frequency or intensity of the changes.

### 3.3 The kidney

The kidney of intact animals is characterized by the presence of elongated hemocyte islets, which are located within the hemocoelic sinuses responsible for draining blood from this organ (**Supplementary Figure S5**). These islets are considered “constitutive” because their presence is not contingent on the existence of an ongoing infection. However, they are also capable of reacting to intruders (17).

#### 3.3.1 The hemocyte islets reactions

**Figure 5:**
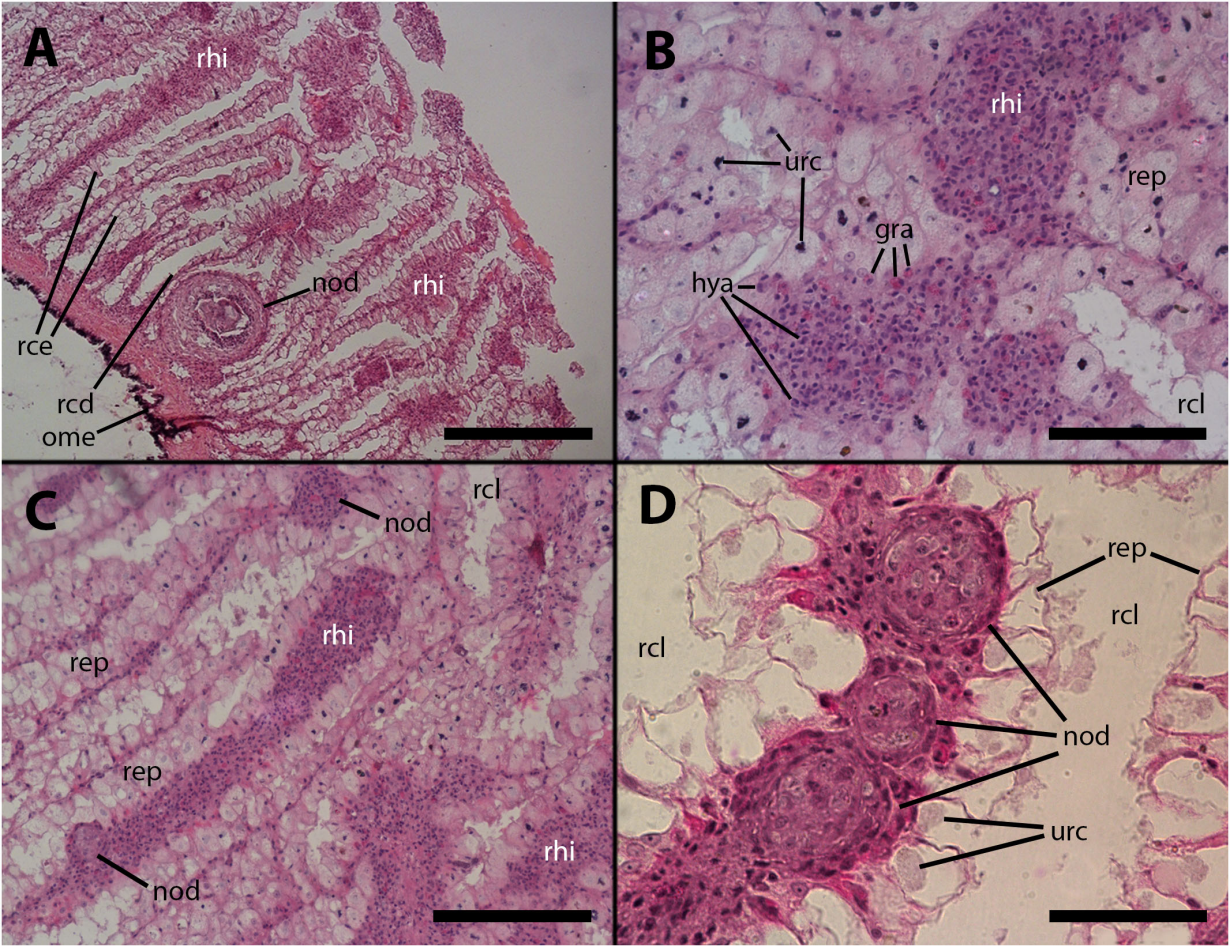
Renal hemocyte islets after *M. marinum inoculation.* **(A)** Enlarged hemocyte islets and a large, apparently regressing nodule (for higher magnification see **Figure 5A**). **(B)** Enlarged islets exhibiting abundant granulocytes intermingled with the predominant hyalinocytes. **(C)** An elongated islet with abundant granulocytes and a small nodule on its surface (white arrowhead). **(D)** Three small nodules in a row near the outflow into the greater efferent renal vessel; granulocytes in the surrounding tissue seem converging toward the nodules (see also **Supplementary Figure S2**); notice the density variation of the urinary concretions within epithelial cells, which is an unexplained phenomenon also found in intact animals. hya, hyalinocytes; gra, granulocytes; nod, hemocyte nodules; ome, outer mantle epithelium; rcd, renal cryptal de-epithelization; rce, renal cryptal epithelium; rcl, renal cryptal lumen; rep, renal epithelium; rhi, renal hemocyte islet; urc, urinary concretions. Treatments: **(A–C)**: inoculation with 65 M CFU; **(D)** inoculation with 100 M CFU. All animals were sacrificed 9 days after inoculation. Scale bars: **(A)** 200 µm; **(B)** 50 µm; **(C)** 100 µm; **(D)** 50 µm.

Vehicle injection does not lead to changes in the renal hemocyte islets. However, upon inoculation with *M. marinum*, the islets undergo modifications in size and the formation of nodules. These nodules can grow to significant dimensions, reaching 200–300 µm in diameter (e.g., **Figure 5A**). The nodules can exhibit either a uniform cell composition (consisting of hyalinocytes and some granulocytes) with no distinct regions, as depicted in **Figures 5B, C**, or a core with a variable cell composition (similar to what is found in intravascular cell aggregations), surrounded externally by a layer of flattened cells. The cores of the nodules comprise, along with mature hyalinocytes and granulocytes, dedifferentiated cells similar to those found in the lung veins and gill epithelium, which are presumed to have the ability to proliferate. Additionally, phagocytic cells, dying cells, and remnants of dead cells are present in the core (**Figure 5A**). Also, acid-fast bacilli and clumps, which are likely mycobacterial remnants, were detected by the Ziehl-Neelsen’s method in hemocyte nodules grown within the renal islets (**Figure 2C**).

**Figure 6:**
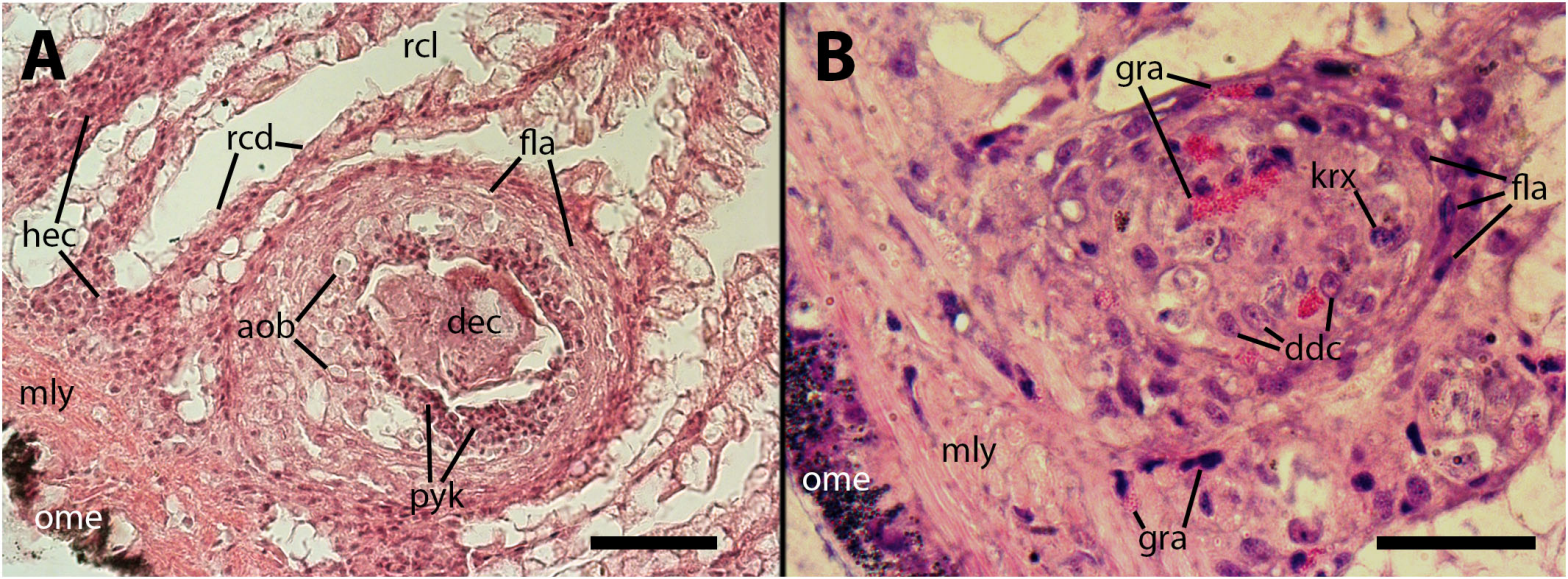
The cores of two subpallial nodules in the kidney demonstrate the variety of cells present within these reactive structures. **(A)** A large, apparently regressing nodule exhibits a central mass composed of dead cells, with surrounding brown bodies similar to those found in the gill epithelium, which are the outcome of hemocytes’ death; adjacent to the central mass, there is a band of small cells with eosinophilic cytoplasm and dark nuclei (presumably dying cells) and, in close proximity, there is evidence of hemocyte congestion around de-epithelized crypts (see also **Figure 9**). **(B)** A medium-sized nodule with conspicuous granulocytes at its core, together with cells with large cytoplasm and euchromatic and nucleolated nuclei (presumably dedifferentiated cells). Several, less conspicuous granulocytes are seen outside the nodule. aob, anucleated ovoid bodies; ddc, dedifferentiated cells; dec, dead cells; fla, flattened cells; gra, granulocytes; hec, hemocyte congestion; krx, karyorrhexis; ome, outer mantle epithelium; rcd, renal cryptal de-epithelization; rcl, renal cryptal lumen; mly, muscle layer; pyc, pyknotic cells. Treatments: **(A)**, 9 days after inoculation with 65 M CFU. **(B)**, 9 days after inoculation with 100 M CFU and the tissue sample was embedded in resin. Scale bars: **(A)** 50 µm; **(B)** 25 µm.

Furthermore, nodules with similar cores can also be found subpallially, which do not appear to originate from the constitutive islets but rather from circulating hemocytes that become trapped in this region where the blood flow is redirected towards the hemocoelic spaces that drain blood from this organ (**Figures 5A**, **6A**). Two subpallial nodules in **Figures 6A, B** exhibit some of the cell types commonly found in the cores of nodules, and some of these cell types resemble those observed in the intravascular aggregations in the lung (**Figure 1A**).

It appears that hemocyte nodules undergo a dynamic process of formation and regression within relatively short time periods. This phenomenon seems to occur in all sites where nodules are formed, particularly in the kidney. Consequently, establishing a clear progression of nodular lesions based on days after inoculation was not possible, as both newly forming and regressing nodules were observed in all groups. Also, no obvious differences were observed between animals inoculated with either of the two doses. However, the time elapsed since inoculation was indeed significant in the case of lung storage tissue decay (Section 3.2.2) and renal epithelial lesions (Section 3.3.2), where the lesions were more pronounced 28 days after inoculation, and there were no signs of recovery during the period of observation.

**Figure 7:**
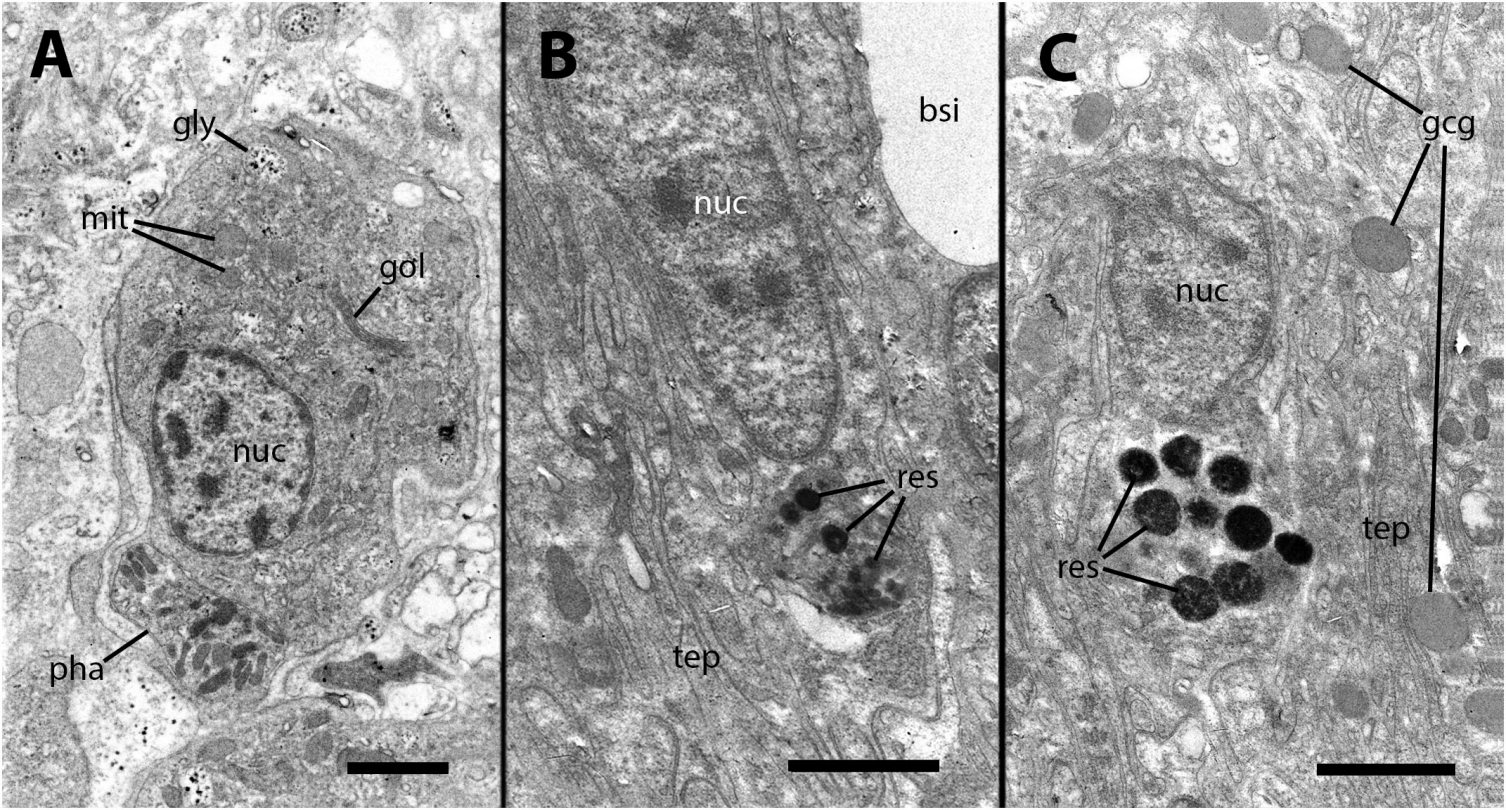
Ultrastructural images of hyalinocytes in the boundary zone between the renal epithelium and hemocyte islets after inoculation. **(A)** A hyalinocyte containing mycobacteria in various stages of digestion within a large phagosome. **(B)** A hyalinocyte surrounded by long, finger-like projections of epithelial renal cells and separated from a hemocoelic sinus by the basal cytoplasm of a renal epithelial cell. Adjacent to the nucleus, a cluster of small and electron-dense spheroidal bodies, likely corresponds to digested mycobacteria. **(C)** Another hyalinocyte with a cluster of electron-dense spheroids corresponding to digested mycobacteria is adjacent to the nucleus and is surrounded by renal epithelial projections. In the vicinity, large round granules of moderate electron density belong to glial cells. bsi, blood sinus; gcg, glial cell granules; gly, glycogen; gol, Golgi stack; mit, mitochondria; nuc, cell nucleus; pha, phagosome; res, residual bodies; tep, thin epithelial projections. Treatment: 9 days after the inoculation with 100 M CFU. Scale bars: **(A–C)** 2 µm.

**Figure 8:**
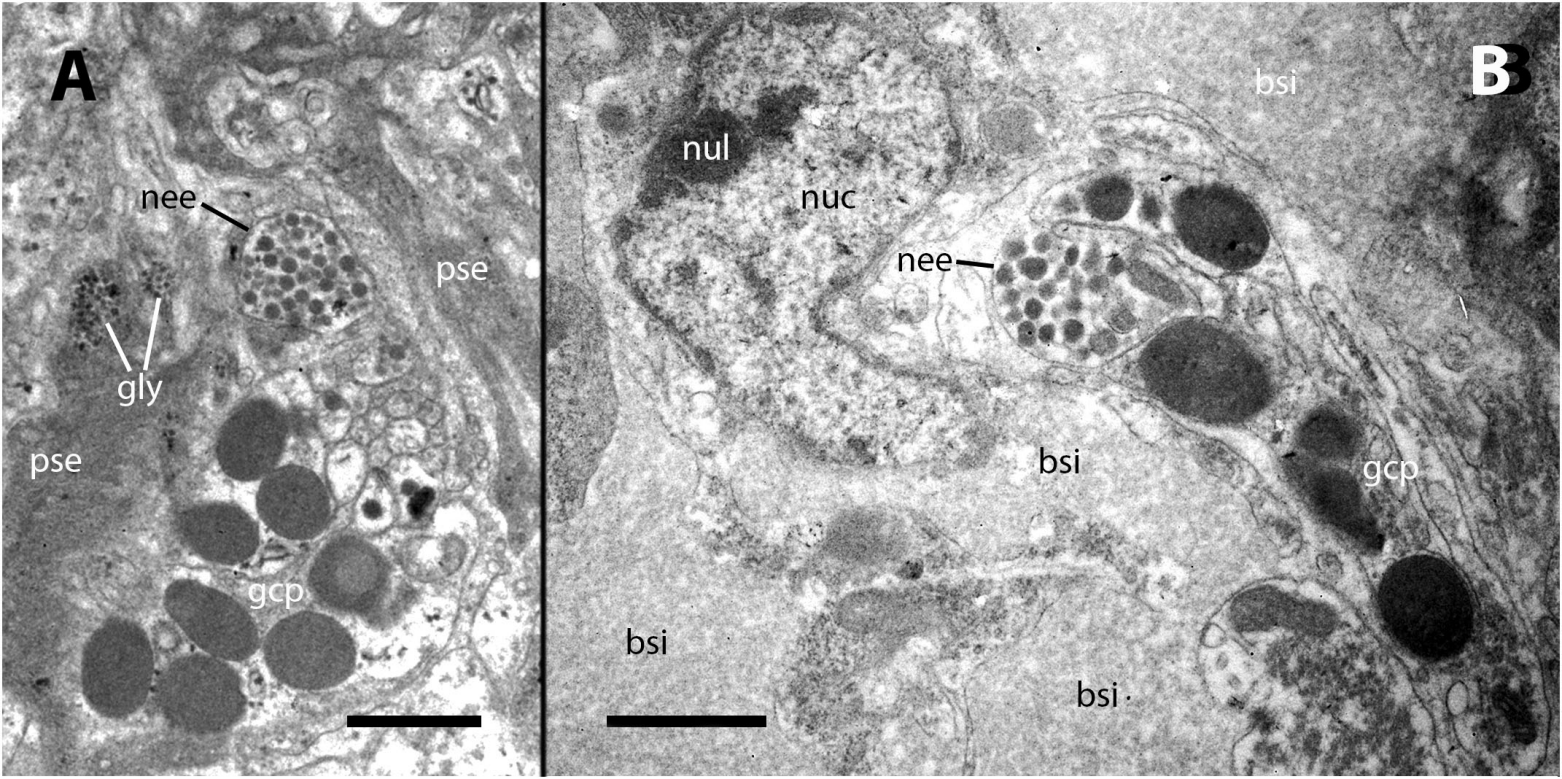
Neurites and glial cells after inoculation. **(A)** A neurite ending in the vicinity of hemocyte pseudopodial extensions is filled with synaptic-like vesicles of varying electron density and is accompanied by glial cells with their typical large granules. **(B)** A neurite ending close to the nucleus of a hyalinocyte located at the surface of a renal islet. bsi, blood sinus; gcp, glial cell process; gly, glycogen; nee, neurite endings; nuc, cell nucleus; nul, nucleolus; pse, pseudopodia. Treatment: Scale bars: **(A, B)** 1 µm.

Our electron microscopy observations were directed to the boundary region between the external surface of the renal hemocyte islets and the basal thin cytoplasmic projections of the renal epithelium. This region is surrounded by blood spaces and encompasses the part of the islets where phagocytosis most frequently occurs (18). Large phagosomes containing phagocytized bacteria were observed in the hemocyte cytoplasm (**Figure 7A**). In other cases, clusters of predominantly round electron-dense bodies were found in close proximity to the nucleus. These electron-dense bodies are interpreted as residual bodies resulting from the digestion of mycobacteria (**Figures 7B, C**). Perhaps, the clumps of acid-fast material observed within the nodules under light microscopy (**Figure 2C**) may be correlated with the phagocytized bacteria seen under transmission electron microscopy. Furthermore, it is noteworthy that numerous neurites accompanied by granule-bearing glial cells frequently terminate in the renal islets of inoculated animals, in close proximity to the nuclei or the cytoplasmic projections of hemocytes (**Figures 8A, B**).

#### 3.3.2 Hemocyte congestion and epithelial loss in subpallial ends of crypts

**Figure 9:**
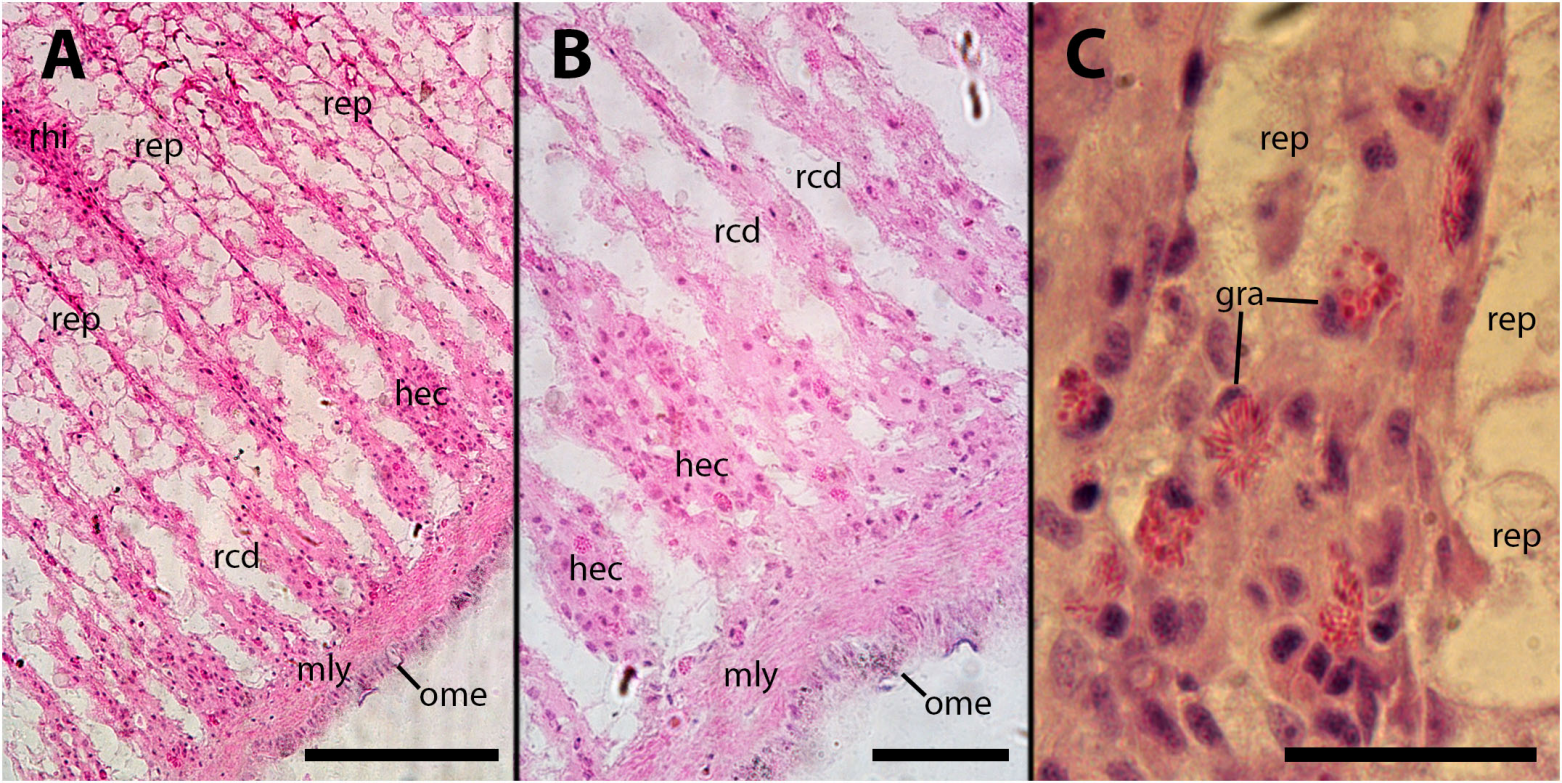
Subpallial end of renal crypts. Hemocyte congestion around the pallial end of renal crypts is accompanied by de-epithelization, 28 days after inoculation of 65 M CFU. gra, granulocytes; hec, hemocyte congestion; mly, muscle layer; ome, outer mantle epithelium; rcd, renal cryptal de-epithelization; rep, renal epithelium, rhi, renal hemocyte islet. Scale bars: **(A)** 150 µm; **(B, C)** 50 µm.

In the subpallial sinus of the kidney, where blood flow stagnates and reverses its direction towards the major draining veins in the ceiling of the kidney chamber (**Supplementary Figure S5**), a noticeable accumulation of hemocytes was already observed 4–9 days after inoculation. This accumulation is accompanied by a reduction in the height of epithelial cells or, in some cases, the complete disappearance of epithelial cells. While a certain degree of de-epithelization may occasionally occur in vehicle-injected animals, it is consistently more pronounced in inoculated animals. In severe cases, there is a complete loss of epithelial cells, with only densely packed groups of hemocytes remaining in the former intercryptal spaces (**Figures 9A, B**). Hemocyte congestion around the pallial end of renal crypts persists 28 days after inoculation, and de-epithelization of the renal crypts becomes more pronounced (**Figure 9C**).

### 3.4 The digestive gland

**Figure 10:**
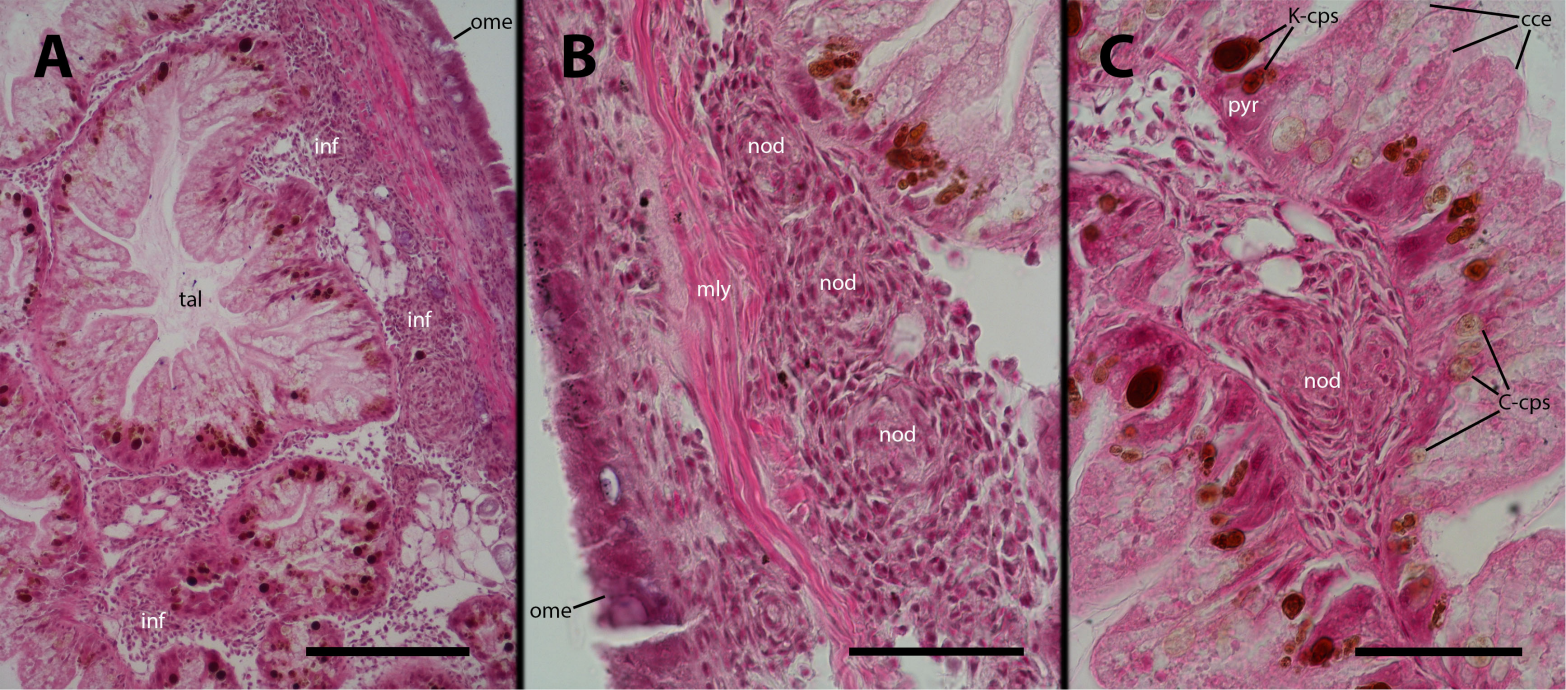
Digestive gland after inoculation. Filling by hemocytes and some nodule formation in interstitial spaces of the digestive gland, 9 days after inoculation with 100 M CFU. C-cps, C morph; K-cps, K morph; cce, columnar cells; inf, hemocyte infiltration; nod, hemocyte nodules; ome, outer mantle epithelium; pyr, pyramidal cell; tal, tubuloacinar lumen. Scale bars: **(A)** 150 µm; **(B)** 50 µm; **(C)** 50 µm.

We examined the digestive gland because the inoculation was performed in the hemocoelic sinuses of this organ (i.e., the visceral sinus). Although we did not observe infiltration in the strict sense (as shown in **Figure 3**), we found that hemocytes separated the outer mantle epithelium from the underlying muscle layer and also occupied the spaces between the tubuloacini, forming either masses or small nodules (**Figures 10A–C**). It is important to note that this occupation by hemocytes may displace some tubuloacini, but it does not result in any significant distortion of the organ’s structure or the tubuloacini in particular.

## 4 Discussion

In this study, we investigated the pathogenesis induced by experimental inoculation with *Mycobacterium marinum* in the lung, gill, and kidney of *Pomacea canaliculata*. Furthermore, we also examined other tissues and organs, including the storage tissue of the lung wall and the digestive gland.

*Pomacea canaliculata* hemocytes, as those of some other gastropods (19, 20), exhibit *in vitro* a notable tendency to approach toward one another, resulting in the formation of aggregates. This phenomenon appears to be a fundamental characteristic of hemocyte behavior in *P. canaliculata*, as evidenced by the formation of spheroidal hemocyte nodules in primary cultures (21). Exploration of their inner structure disclosed a central core surrounded by lacunae, and an outer layer of flattened cells. Furthermore, floating hemocytes in culture were observed to attach to the outer surface of these nodules, where they integrated with the flattened cell layer (21).

In the open circulatory system of gastropods, such as in ampullariids, the characteristics of the hemocoel bed can vary significantly across different regions, which has a direct impact on the behavior of hemocytes. The presence of a lung in ampullariids further adds complexity to the hemocoel bed compared to other caenogastropods. As a result, hemocytes have a tendency to aggregate in some regions of slow blood flow or in other narrow hemocoel spaces, driven by both mechanical constraints and their own behavior.

After mycobacterial inoculation, these aggregations give rise to floating hemocyte nodules (**Figures 1A, B**), which resemble the nodules observed in culture and can be quite large. However, when these nodules obstruct hemocoelic spaces or become anchored to the vascular wall, they can extend into the surrounding tissues. In the case of the lung, this can lead to the obstruction of extensive regions of the respiratory lamina (**Figures 1C, D**), compromising air respiration, which is crucial for an amphibious snail like *P. canaliculata* (22, 23). Additionally, tissue infiltration may occur due to attraction to decaying tissues or dying cells (**Figure 3**).

However, significant pathological changes occur in the core of nodules in response to the infection, and not in nodules grown in sterile cultures. The cellular composition within the core of nodules in inoculated animals can be categorized into three main types (1): mature hyalinocytes and granulocytes (2), dedifferentiated hemocytes that are likely able to undergo proliferation and differentiation into newly mature hemocytes, and (3) phagocytic hemocytes characterized by a nucleus showing moderate chromatin condensation and a large cytoplasm (see **Figures 1**, **4**, **5**, **6**). Additionally, the core of the nodules may contain anucleated masses exhibiting varying degrees of basophilia/eosinophilia, which are likely remnants of dead cells (**Figure 6A**). Also, pigmented ovoid bodies may replace intraepithelial granulocytes, indicating the occurrence of cell death (**Figures 4A, B**). This cell death could be attributed to oxidative damage, leading to the deposition of lipofuscin (e.g., 24). Occasional formation of small nodules in the gill hemocoel may also occur (not shown in pictures).

Hemocyte nodules are formed on the surface of the constitutive hemocyte islets of the kidney, or within the islets themselves (**Figures 5A–D**). These islets play a central role in the immune system of *P. canaliculata*, serving as a hematopoietic organ, a phagocytic barrier, and a potential reservoir for hemocytes (5, 17). And there is also evidence of a possible neural control (**Figure 8**). In addition to the nodules associated with the constitutive hemocyte islets, there is hemocyte congestion around the pallial end of renal crypts (**Figure 9**). This congestion likely occurs due to a slowdown in blood flow when the blood enters the draining hemocoelic spaces and redirects itself toward the large draining vessels located along the ceiling of the renal chamber. Though the hemocytes are sometimes disorganized here, they may also form nodules (**Figures 6A, B**). As also happens in the lung, the core of the nodules in the kidney contains both differentiated and dedifferentiated hemocytes, as well as large phagocytic cells and remnants of dead cells (**Figure 6B**).

The stagnation of blood flow in the subpallial region leads to a reduction in local oxygen tension, resulting in a condition known as “stagnant hypoxia.” This phenomenon has long been recognized in humans (25). In *P. canaliculata*, this condition may contribute to the death of renal epithelial cells (**Figure 9**). The involution of this part of the urinary system can affect the resorption of solutes from the primary urine, which is formed by ultrafiltration in the heart auricle (26–28) and, consequently, it may lead to renal dysfunction.

Though it has been assumed that nodules could be a common defense strategy for restraining the penetration of intruders in a wide variety of metazoans (5), Ramakrishnan (29) proposed that granulomas (i.e., nodules) could also serve as a means for the intruders’ perpetuation within the host. In fact, her proposition was based on observations of transparent zebrafish larvae, where infected macrophages entered and exited the nodules, thereby dispersing the mycobacteria outside them (30). In line with this, Cueto et al. (21) employed time-lapse video microscopy to capture the dynamic ingress and egress of hemocytes from the aggregations in sterile cultures and found similar results.

The periphery of renal islets is the region where particles are most actively phagocytized in *P. canaliculata* (18). Hemocytes in this area establish close contact with long finger-like projections extending from the basal domain of renal epithelial cells (5, and **Figures 6B, C**). Therefore, we directed to this area our observations under transmission electron microscopy and, in agreement with those previous findings, we found that the hemocytes form islets when retained and intermingled with the finger-like projections of renal epithelial cells. Some clusters of electron-dense spherical bodies that are concretions of much smaller particles are observed near the hemocytes’ nuclei (**Figures 7B, C**). These bodies are the likely candidates for being digested mycobacteria within hemocytes in renal islets.

In addition to the aforementioned findings, the presence of glial cell-coated neurite endings on the surface of the hemocyte islets may represent the first reported evidence of a neuro-immune interface in a caenogastropod (**Figure 8**). Possibly, these endings may be influencing the proliferation or the migration of hemocytes into and out of these islets. The delivery of hemocytes from reservoirs to the circulation may be crucial for a rapid immune response against bacterial intruders, and the renal islets are likely to play a pivotal role in this process.

Uric acid plays a crucial role in the antioxidant defense system of *P. canaliculata* during arousal from estivation and hibernation (13, 14, 31). It accumulates in a widespread storage tissue system that includes the lung wall (11, 15). During resumption of full aerobic respiration, when oxidative stress results, the accumulated uric acid acts as a scavenger of reactive oxygen species (ROS), providing protection to the snail’s tissues against oxidative damage (13, 14, 31). Ultrastructural changes in intracytoplasmic crystalloids, which indicate the storage and release of uric acid, have also been observed in active normometabolic animals, suggesting that uric acid plays a role in maintaining oxidative balance even in the resting state (15). Therefore, the decay of the storage tissue in parts of this system in inoculated animals (**Figure 3**) can be attributed to oxidative stress-induced consumption (13, 14, 31). Furthermore, one may speculate that an increased generation of ROS could be also a result of the infection. In line with this, there has been shown that the oxidative status may play a significant role in the formation and expansion of mycobacterial granulomas (32).

As expected, the digestive gland, where *M. marinum* was administered into the hemocoel, exhibited a massive presence of hemocytes and the formation of nodules in the spaces between the tubuloacini. However, no consistent changes where observed in the tubuloacini themselves, whose cells harbor two morphs of a putative endosymbiont, contained within specific cells: the so-called C-morphs are found in the columnar cells, while the K-morphs are found in the pyramidal cells (33, 34). However, this structural arrangement remained unaffected despite the loads of hemocytes in the interstitial spaces.

May this experimental infection result in an experimental disease paralleling the chronicity of tuberculosis in humans or other animals? Ramakrishnan proposed for the zebrafish (29) that nodules would serve a dual role, both sequestering the pathogens and providing a shelter for them, and this may partly explain the chronicity of the disease, as the nodules can periodically release the pathogens, contributing to re-infections over time. The same idea may apply to the short, dynamic cycle of nodule formation and regression apparently occurring in *P. canaliculata* (Section 2.2.1) and may also provide an explanation for the occurrence of newly formed nodules and other hemocyte aggregations weeks after a single inoculation to *P. canaliculata*. This snail has also shown a notable resilience to *M. marinum* as a pathogen, which may be linked, though in an unprecise way, to the robustness of the species. But a related and intriguing question is what happens with the large doses of *M. marinum* that fall below the threshold for eliciting immune cell reactions consistently (65 M CFU per ~15 g of soft tissues in an adult *P. canaliculata*)? There is a possibility that the pathogens are effectively neutralized by soluble factors that would be present in the host, as it has been extensively studied in heterobranch gastropods (e.g., 35). Soluble factors such as lectins, complement proteins, antimicrobial peptides, and others may indeed play a significant role in the defense mechanisms of Caenogastropoda, including ampullariids and specifically *P. canaliculata*. Much research would be needed for comprehending the involvement of these soluble factors in the defense mechanisms of these gastropods.

The cellular responses exhibited by *P. canaliculata* to *M. marinum* infection make it a suitable species for studying mycobacterial infections and their associated cellular and physiological effects. Furthermore, *P. canaliculata* is a robust and easily bred gastropod, with a wealth of available fundamental information spanning molecular biology, physiology, behavior, ecology, and morphology (8, 36). Importantly, there is also a solid foundation of knowledge regarding its cellular immunobiology, including the identification of different hemocyte types (12, 37) and the localization of hematopoiesis (5, 38). Additionally, the distinct tendency of *P. canaliculata* hemocytes to form spheroidal aggregates resembling those seen in granulomatous diseases, has been observed both *in vivo* and *in vitro* (21). This comprehensive background knowledge makes *P. canaliculata* a potentially excellent model for investigating mycobacterial infections and their underlying mechanisms.

## Data availability statement

The original contributions presented in the study are included in the article/**Supplementary Material**. Further inquiries can be directed to the corresponding author.

## Ethics statement

The animal study was approved by Institutional Committee for the Care and Use of Laboratory Animals (School of Medicine, National University of Cuyo). The study was conducted in accordance with the local legislation and institutional requirements.

## Author contributions

Conceptualization, AC-V and CR. Methodology, CR, CC-F, and AC-V. Investigation, CC-F, CR, CG, and AC-V. Writing—original draft preparation, AC-V. Writing—review and editing, AC-V, CR, CC-F, CG, and IAV. Visualization, CC-F and CR. Supervision, AC-V and CR. Project administration, CR, IAV and AC-V. Funding acquisition, CR, IAV, and AC-V. All authors contributed to the article and approved the submitted version.
