# Supplementary figures and images for: Pathogenesis of an experimental mycobacteriosis in an apple snail
Source: Front Immunol. 2023 Oct 9;14:1253099. doi: 10.3389/fimmu.2023.1253099 (PMC10593440; doi:10.3389/fimmu.2023.1253099)

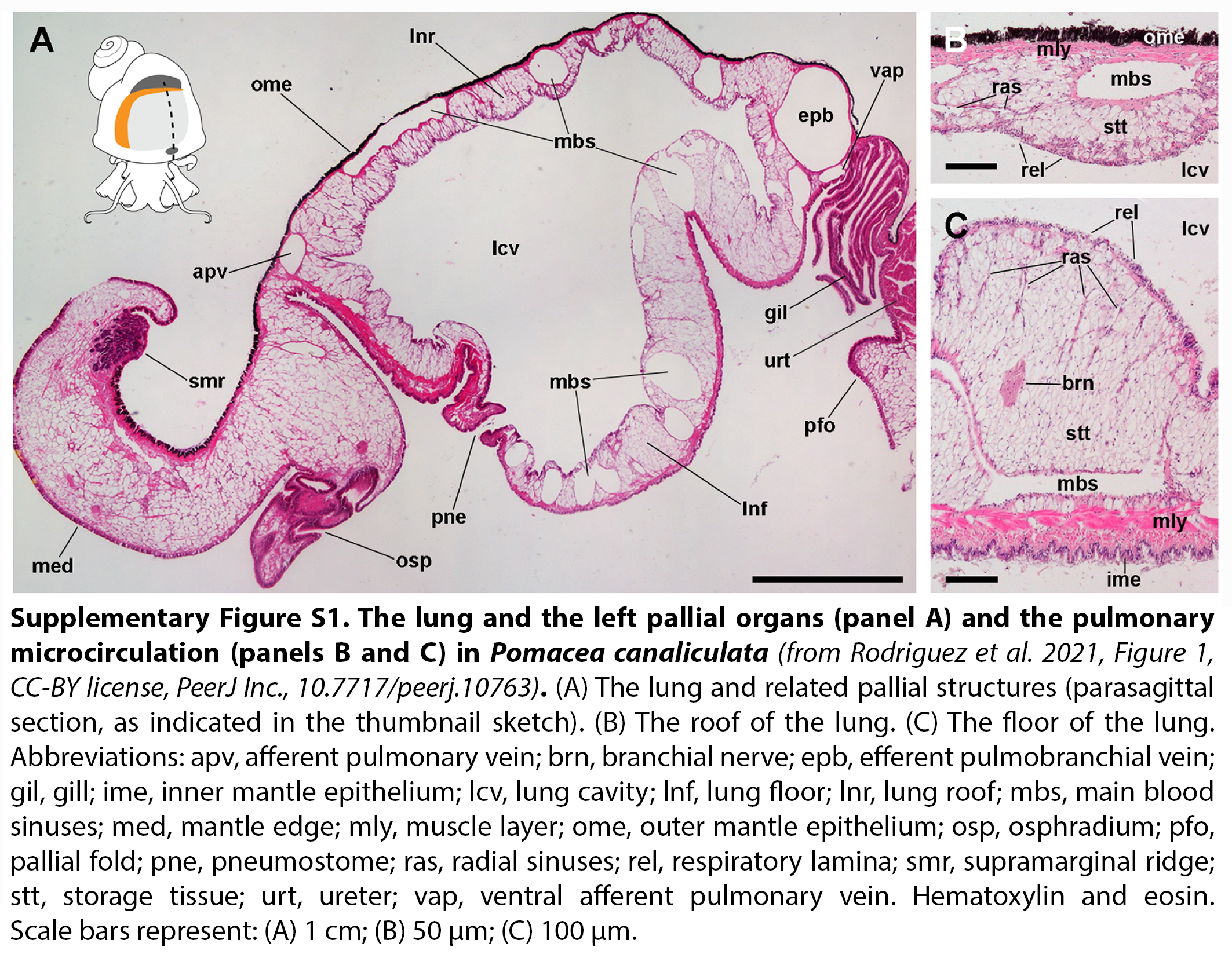

Supplement: Supplementary file 1 [file Image_1.tif]

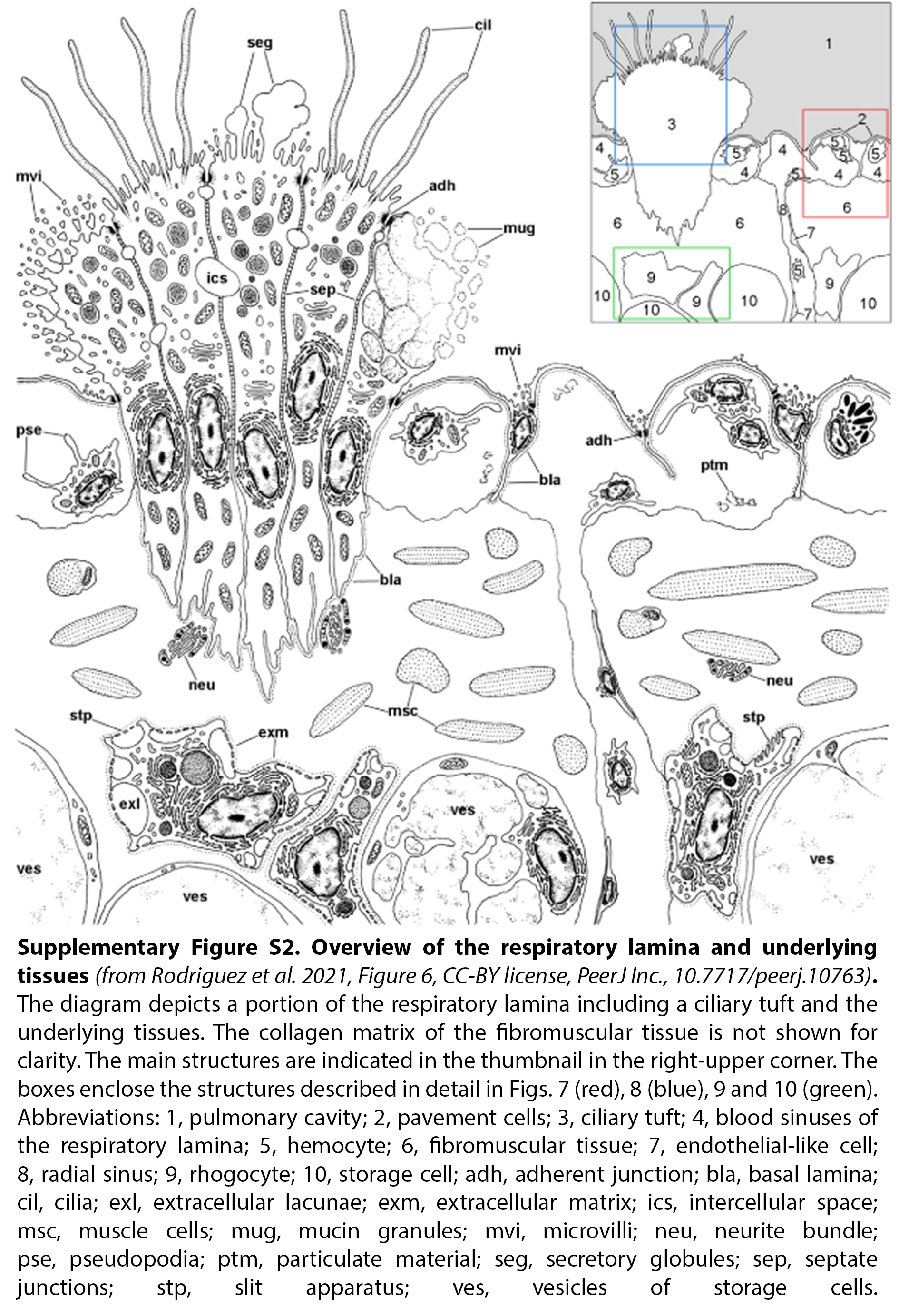

Supplement: Supplementary file 2 [file Image_2.tif]

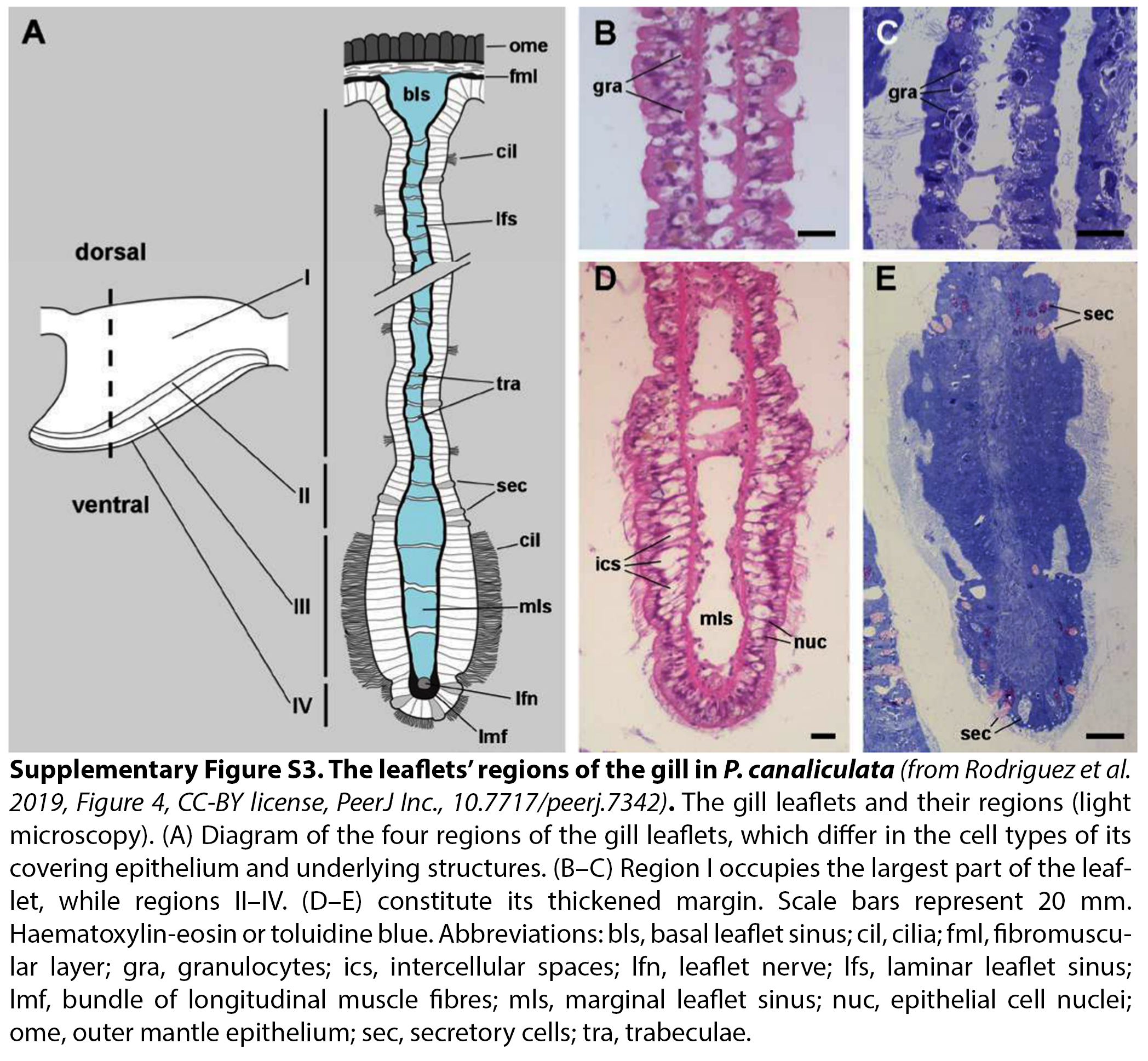

Supplement: Supplementary file 3 [file Image_3.tif]

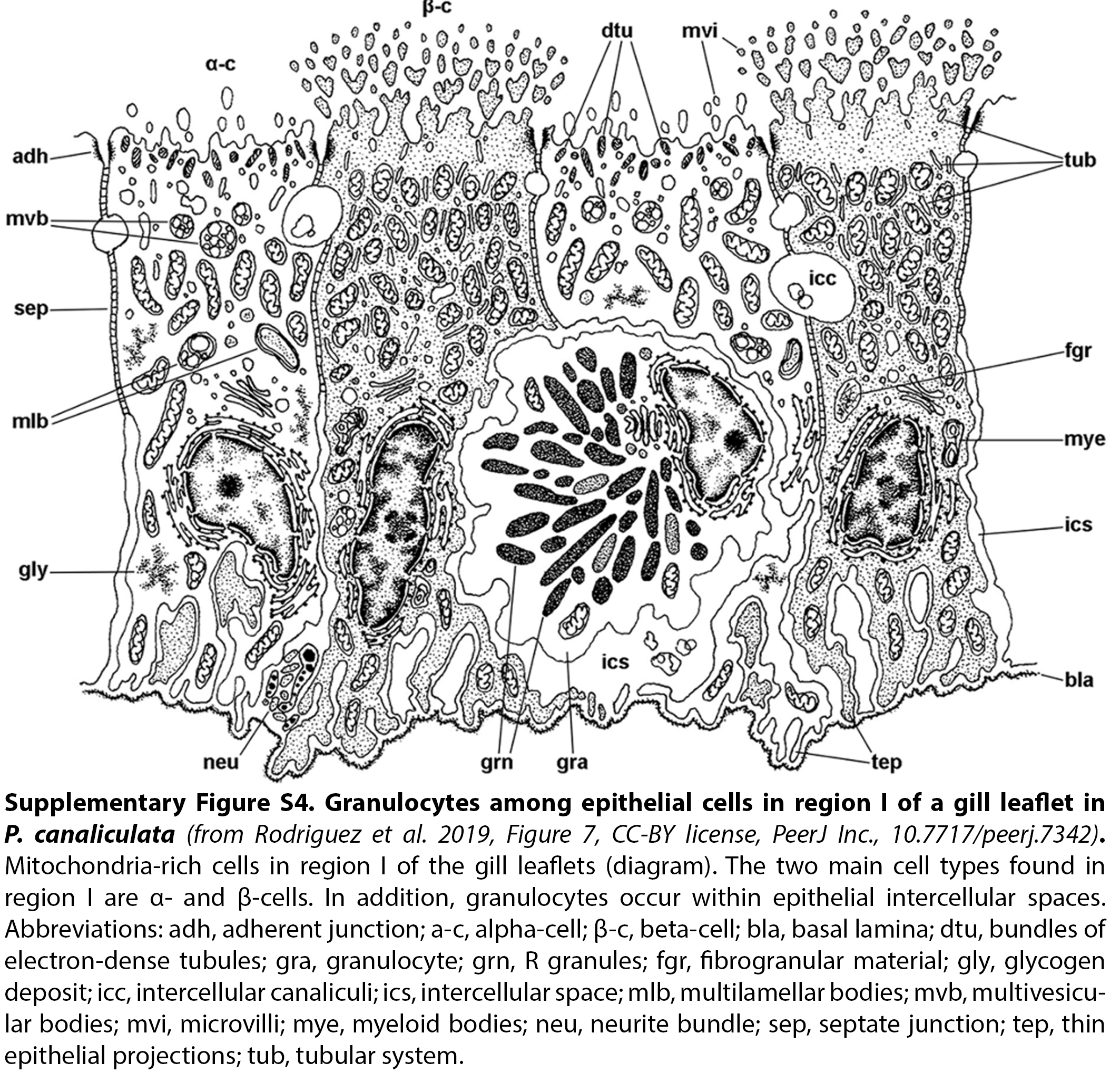

Supplement: Supplementary file 4 [file Image_4.tif]

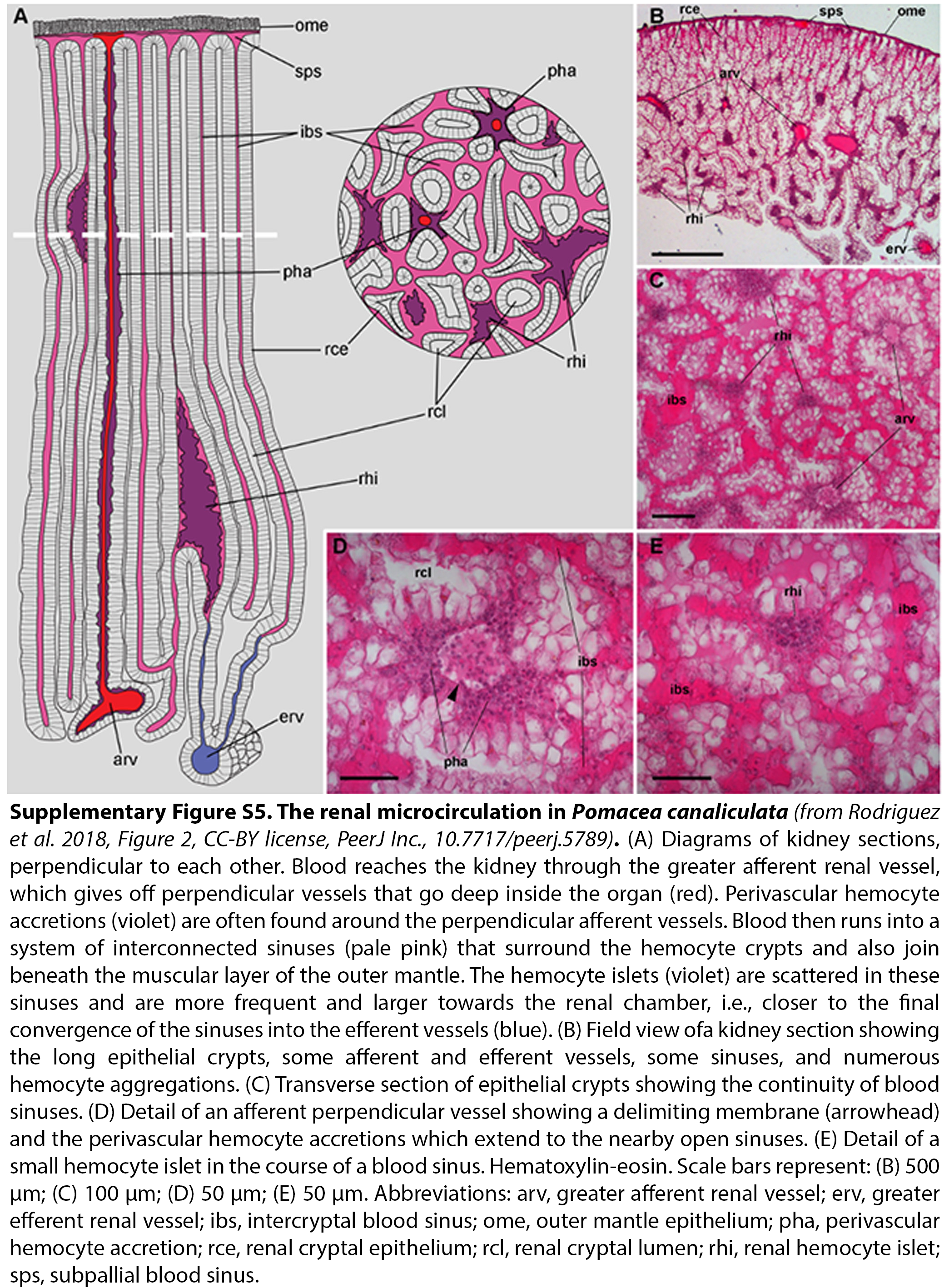

Supplement: Supplementary file 5 [file Image_5.tif]
